# Swept-source OCT in patients with multiple evanescent white dot syndrome

**DOI:** 10.1186/s12348-018-0159-2

**Published:** 2018-10-13

**Authors:** Felipe Pereira, Luiz H. Lima, Alexandre Gomes B. de Azevedo, Claudio Zett, Michel E. Farah, Rubens Belfort

**Affiliations:** 1 Department of Ophthalmology, Federal University of São Paulo, Rua Botucatu, 821, Vila Clementino, São Paulo, SP 04023-062 Brazil; 2 Vision Institute, São Paulo, Brazil; 3 Pontificia Universidad Católica de Valparaíso, Valparaíso, Chile

**Keywords:** Choriocapillaris, MEWDS, Outer retina, Swept-source OCT

## Abstract

**Background:**

Swept-source optical coherence tomography (SS-OCT) has a higher scanning rate and longer wavelength in comparison with spectral-domain OCT (SD-OCT), allowing an improved imaging of retinal vascular plexuses and choriocapillaris. The present two patients diagnosed with multiple evanescent white dot syndrome (MEWDS) underwent fundus autofluorescence (FAF), en-face SS-OCT, and SS-OCT angiography (OCTA) imaging, and its features were described and correlated.

**Results:**

The clinical and imaging findings of both cases were consistent with the diagnosis of MEWDS. Color fundus photograph revealed subtle deep retinal white spots in the posterior pole and around the optic disk. FAF showed several hyperautofluorescent lesions corresponding topographically to the subtle deep retinal white lesions observed on color fundus photographs. Cross-sectional SS-OCT showed disruption of the ellipsoid zone (EZ) within the macular area in all study patients. En-face SS-OCT at the level of the outer retina showed lower reflectivity correspondent to the diffuse attenuation due to the EZ disruption on cross-sectional OCT. SS-OCTA demonstrated flow preservation within the retinal vasculature and choriocapillaris.

**Conclusions:**

SS-OCT imaging allows a better visualization of the choriocapillaris, and its normal appearance in MEWDS may suggest that the outer retina and photoreceptors represent the primary site of inflammation.

## Background

Multiple evanescent white dot syndrome (MEWDS) was first described by Jampol et al. [1] in 1984 as a transient chorioretinal disease of unknown etiology and typically affects young healthy women who present with decreased visual acuity, photopsia, and enlarged blind spot. MEWDS is unilateral in 80% of the cases, and small white dots in the retinal fundus associated with a granular fovea represent the most characteristic features of this disease. Other less common fundus features include an edematous appearing optic disc and the presence of cells in the vitreous. The majority of MEWDS patients present a spontaneous resolution of all lesions and improvement in visual acuity within weeks [2, 3].

Although the precise pathogenesis of MEWDS is unknown, an infectious etiology is thought to be involved in the pathogenesis of the disease since usually a viral prodrome exists [1–3]. The multimodal imaging analysis is very important to study patients with MEWDS because its clinical appearance may be indistinct from other white-dot syndromes. The imaging hallmarks of the disease include punctate hyperfluorescence in a wreath-like pattern of dots and optic disc leakage on fluorescein angiography (FA), widespread nummular hypofluorescent dots on indocyanine green angiography (ICGA), hyperautofluorescent lesions on fundus autofluorescence (FAF), and disruption of outer retina, specifically in the ellipsoid zone (EZ) on spectral-domain optical coherence tomography (SD-OCT) and en-face OCT [4–6].

First analysis of late hypofluorescent lesions with ICGA imaging in MEWDS led to the conjecture that the disease might be due to some impairment in choriocapillaris flow [7]. However, current reports using SD-OCT have implied the outer retina as the site of initial defects in MEWDS [6, 8]. Because swept-source OCT (SS-OCT) has a higher scanning rate and longer wavelength in comparison with SD-OCT, it allows an improved imaging of retinal vascular plexuses and choriocapillaris [9, 10]. In the present report, two patients diagnosed with MEWDS underwent SS-OCT to study the outer retina and choriocapillaris features.

## Case report

### Case #1

**Fig. 1:**
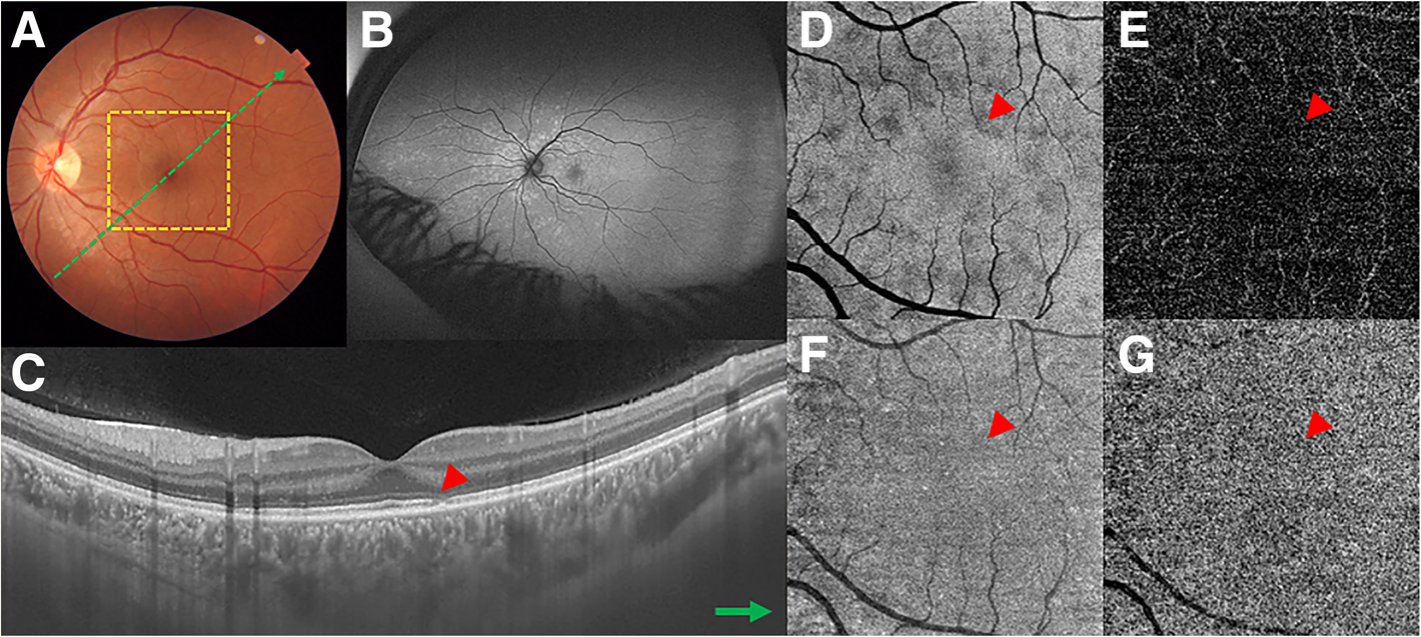
Case #1. Multimodal imaging of a 28-year-old woman with multiple evanescent white dot syndrome (MEWDS). **a** Color fundus imaging shows discrete white lesions in the macular area. **b** Wide-field fundus autofluorecence (FAF) depicts a peripapillary hyperautofluorescent area associated with widespread hyperautofluorescent lesions in the retinal fundus. **c** Swept-source cross-sectional optical coherence tomography (OCT) represented by the green line in image **a** shows disruption of the ellipsoid zone (EZ) (red arrowhead). **d** En-face OCT at the level of the outer retina shows hyporeflective spots that corresponded to the EZ disruption on cross-sectional OCT (red arrowhead). **e**–**g** OCT angiography (OCTA) of outer retina (**e**), en-face OCT at the level of choriocapillaris (**f**), and OCTA of choriocapillaris (**g**) show homogeneous reflectivity without artifacts or any other abnormalities

A 28-year-old woman presented with unilateral blurred visual acuity associated with photopsias. Approximately 2 days before the visual symptoms presentation, the patient reported flu-like symptoms, such as fever and headache. The patient had an unremarkable previous medical and ocular history, and there was no recent contact with animals, including cats. Laboratory and systemic imaging tests revealed a normal complete blood count (CBC) and chest and sinus X-rays. Serologies for syphilis, cytomegalovirus, herpes simplex virus, human immunodeficiency virus, *Bartonella*, *Histoplasma capsulatum*, *Toxoplasma gondii*, *Toxocara canis*, and *Borrelia burgdorferi* were negative. On ocular examination, the best-corrected visual acuity (BCVA) was 20/20 in the right eye and 20/25 in the left eye. Biomicroscopy of anterior segment, pupillary reactions, and intraocular pressure were normal in both eyes. Color fundus photograph of left eye revealed subtle deep retinal white spots in the posterior pole and around the optic disk. FA demonstrated wreath-like punctate areas of early hyperfluorescence that corresponded to the deep white retinal lesions. Optic disc staining was observed in the late phase of FA. FAF showed several hyperautofluorescent lesions corresponding topographically to the white lesions observed on color fundus photograph. Cross-sectional OCT (DRI Swept Source OCT Triton, Topcon, Japan) demonstrated disruption of the EZ at the same topography of the spots seen on both the FAF and FA. Punctate hypereflective lesions and hypereflective dots were observed in the outer nuclear layer (ONL) and choroid, respectively. En-face OCT at the level of outer retina (DRI Swept Source OCT Triton, Topcon, Japan) showed multiple hyporeflective spots corresponding to the disruption of the EZ seen on the cross-sectional OCT. There was absence of flow impairment in both the retinal and choroidal vasculature on OCTA (Fig. 1). At 6 month follow-up, the MEWDS lesions spontaneously disappeared, and the visual acuity returned to 20/20.

### Case #2

**Fig. 2:**
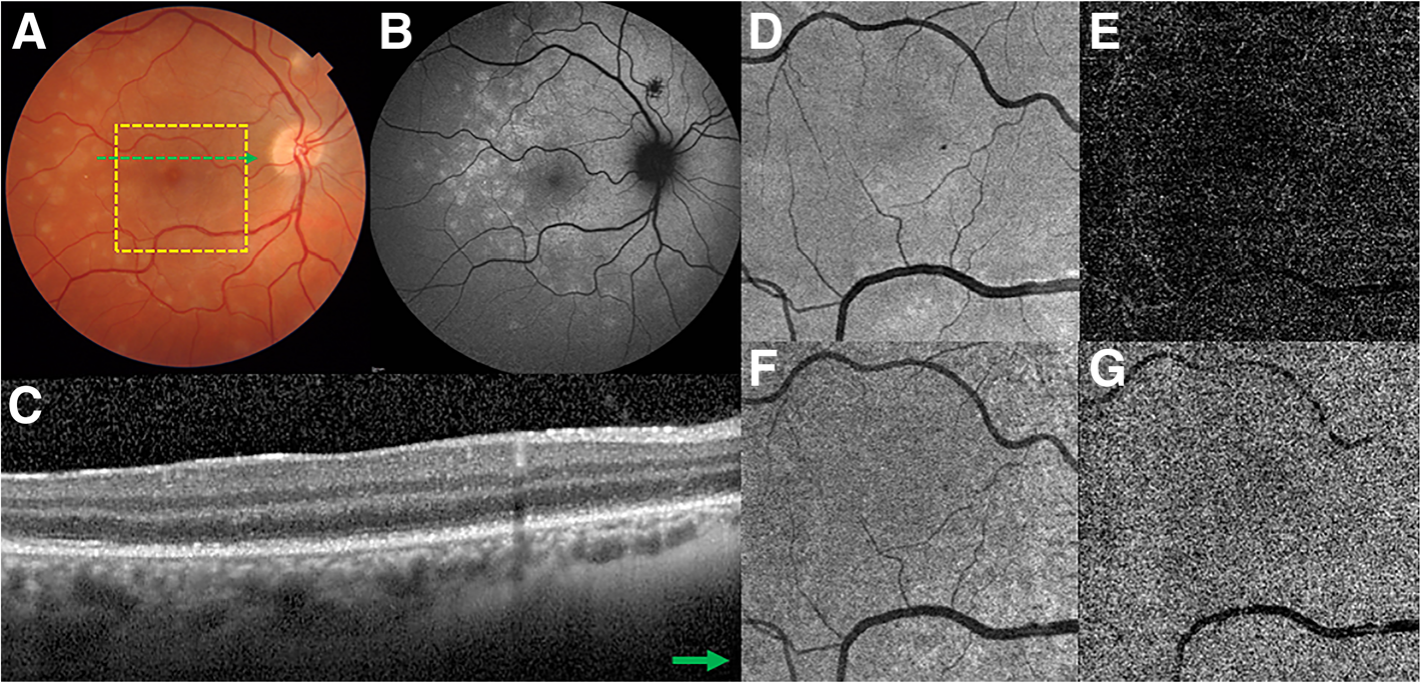
Case #2. Multimodal imaging of a 33-year-old woman with MEWDS. **a** Color fundus imaging shows discrete white lesions in the macular area. **b** FAF depicts a peripapillary hyperautofluorescent area associated with widespread hyperautofluorescent lesions in the retinal fundus. **c** Cross-sectional OCT (represented by the green line in image **a**) shows disruption of the EZ. **d** En-face OCT at the level of the outer retina shows an intrinsic lower reflectivity correspondent to the diffuse attenuation due to the EZ disruption on cross-sectional OCT. **e**–**g** OCTA of outer retina (**e**), en-face OCT at the level of choriocapillaris (**f**), and OCTA of choriocapillaris (**g**) demonstrate homogeneous reflectivity without artifacts or any other abnormalities

A 33-year-old woman presented with unilateral decreased visual acuity associated with photopsias. Approximately 10 days before the visual symptoms presentation, the patient reported flu-like symptoms, such as fever and headache. The patient had an unremarkable previous medical and ocular history, and there was no recent contact with animals, including cats. Laboratory and systemic imaging tests revealed a normal CBC and chest and sinus X-rays. Serologies for syphilis, cytomegalovirus, herpes simplex virus, human immunodeficiency virus, *Bartonella*, *Histoplasma capsulatum*, *Toxoplasma gondii*, *Toxocara canis*, and *Borrelia burgdorferi* were negative. On ocular examination, the BCVA was 20/200 in the right eye and 20/25 in the left eye. Biomicroscopy of anterior segment, pupillary reactions, and intraocular pressure were normal in both eyes. Color fundus photograph of left eye revealed subtle deep retinal white spots in the posterior pole and midperipheral retina. Similar confluent lesions were also observed around the optic disk. FA demonstrated wreath-like punctate areas of early hyperfluorescence that corresponded to the deep white retinal lesions. FAF showed several hyperautofluorescent lesions corresponding topographically to the white lesions observed on color fundus photograph. Cross-sectional OCT (DRI Swept Source OCT Triton, Topcon, Japan) demonstrated punctate hypereflective lesions in the ONL and hypereflective choroidal dots, and disruption of the EZ at the same topography of the spots seen on both the FAF and FA. En-face OCT at the level of outer retina (DRI Swept Source OCT Triton, Topcon, Japan) shows an intrinsic lower reflectivity correspondent to the diffuse attenuation due to the EZ disruption on cross-sectional OCT. There was absence of flow impairment in both the retinal and choroidal vasculature on OCTA (Fig. 2). At 6 month follow-up, the MEWDS lesions spontaneously disappeared, and the visual acuity returned to 20/20.

## Discussion

The diagnosis of MEWDS may be challenging because the lesions are frequently discrete with a characteristic spontaneous resolution [1–3]. The typical MEWDS lesions are observed as deep white spots in the peripapillary and macular areas, according to the natural course of the disease, these lesions disappear and the visual acuity improves to 20/20 during the follow-up period. In the present study cases, the MEWDS lesions were typically hyperautofluorescent and this pattern of FAF may represent increased lipofuscin deposition in the retinal pigment epithelium (RPE) or photoreceptor loss unmasking the natural fluorescence of the underlying RPE [11]. On cross-sectional SD-OCT, disruption of the EZ was observed and corresponded topographically to the hyperautofluorescent spots on FAF. Punctate hypereflective lesions of variable size in the ONL were also observed in our cases. These hypereflective dots in the ONL could be due to lipofuscin migration from the damaged RPE cells into the outer retinal layers or may represent photoreceptor debris shed and accumulated in the outer retina [6, 12]. Another cross-sectional SD-OCT feature was represented by hypereflective submacular choroidal dots that progressed to the classic presentation of EZ disruption within few days. Fiori et al. noticed that 40% of their study patients presented hypereflective dots in the inner choroid close to the site of outer retinal layers disruption. These dots are also observed in other choroiditis conditions, and it is possibly due to focal inflammatory cells aggregate [13].

Previous studies using en-face OCT have described two different patterns of lesions in MEWDS patients. Larger hyporeflective and confluent spots colocalized to the level of the EZ and smaller hypereflective dots colocalized to the ONL [6, 14]. In our cases, the two study patients presented a diffuse reduction of reflectance in the outer retina. This probably happened because the en-face scans were smaller (4.5 × 4.5 mm) and there was EZ and interdigitation zone (IZ) discontinuation in this entire area. Possibly, if a larger OCT scan was used, the difference between the affected outer retina (hyporeflective pattern) and healthy outer retina (hypereflective pattern) would be identified. OCTA is a new technology that allows the visualization of chorioretinal vessels flow without dye injection. Although OCTA does not detect vascular leakage, it can identify fine vascular structures that may have important value in cases of choroidal neovascularization associated with MEWDS [15].

There is still controversy regarding whether MEWDS is primarily an outer retinal or choriocapillaris/choroidal disease. Jampol et al. implicated photoreceptor damage to underlying RPE dysfunction in their first report of disease [1]. ICGA imaging analysis in MEWDS has associated the hypofluorescent lesions with impairment in choriocapillaris flow [7]. There is evidence of increased choroidal thickness in the acute phase of the disease in both the affected and fellow eyes that returns to the normal choroidal thickness in the convalescent phase of the disease [16]. Fiori et al. observed that not only the choriocapillaris layer was thickened, but also the large choroidal vessels layer when compared with the inactive phase or the fellow eye. The author hypothesized that the initial inflammatory process begins in the inner choroid and then spread to the entire choroid and outer retinal layers [13]. Conversely, several studies using SD-OCT have demonstrated that MEWDS starts in the outer retina and the photoreceptors, specifically in the EZ [17–19]. In their MEWDS series, Pichi et al. [6] demonstrated that the choriocapillaris/choroid and superficial and deep retinal capillary networks were comparable to normal healthy controls. Probably, the hypofluorescence observed on ICGA in MEWDS cases is due to tissue staining rather than choriocapillaris/choroidal injury. Using SS-OCT, Yannuzzi et al. [8] also reported flow preservation within the retinal and choriocapillaris vasculature. Similarly, our SS-OCTA images show that choriocapillaris flow appears to be preserved in the early stages of disease. Therefore, this finding should lead to a more specific research into the pathogenesis of MEWDS. Although oral corticosteroids are sometimes prescribed for MEWDS, our study patients did not take any medication during the disease follow-up as MEWDS is habitually self-resolving and clinical therapy is of undefined aid.

The normal appearance of choriocapillary circulation during the acute phase of disease may suggest that the pathologic process in MEWDS is represented by a primary injury in the outer retina and the photoreceptors. Larger series with long-term follow-up is needed to confirm the preservation of choriocapillaris flow on SS-OCTA in patients with MEWDS.

## Abbreviations

BCVA: Best-corrected visual acuity
CBC: Complete blood count
EZ: Ellipsoid zone
FA: Fluorescein angiography
FAF: Fundus autofluorescence
ICGA: Indocyanine green angiography
MEWDS: Multiple evanescent white dot syndrome
OCT: Optical coherence tomography
ONL: Outer nuclear layer
RPE: Retinal pigment epithelium
SD-OCT: Spectral-domain optical coherence tomography
SS-OCT: Swept-source OCT
